# The Code of Ethics*Read before the Chicago Practitioner’s Club, April 25, 1892.

**Published:** 1892-06

**Authors:** E. J. Doering

**Affiliations:** Chicago


For Daniel's Texas Medical Journal.

### THE CODE OF ETHICS.*

BY E. J. DOERING, M. D., CHICAGO.

IN THIS country, where liberty is confounded with unbridled license, when justice is so often an article of merchandise, where statesmanship is supplanted by political trickery, where science is used as a cloak to cover ignorance, where, in short, quackery, humbuggery and criminality flourish like vegetation in the tropics, in such a land there is need for a standard, around which honorable men can gather, and by the strength of union elevate their calling to a higher plane, for their own personal good and for the benefit of the generation to follow. For this reason, gentlemen, I am a firm believer, first, last and all the time in that grand guidon of the medical profession, the code of ethics of the American Medical Association, a document as clear and concise, as comprehensive and adequate as the Declaration of Independence or the Constitution of the United States. Read it as often as you can—the oftener the better, paragraph after paragraph, page after page, and you will be better men, better physicians, better able to cope with the problems and difficulties that daily beset you in your chosen calling. Simple in its precepts, just in its teachings, correct in its doctrines, grand in its conception, I would it were required that every candidate for the degree of Doctor of Medicine commit this code to memory, in lieu of preferring the graduating thesis, a custom as absurd as it is useless.

Antagonism is the price of existence, and the nearer you ap-

*Read before the Chicago Practitioner's Club, April 25, 1892.

proach the ideal the more bitter the opposition. Hence, it is not surprising that of late years there has been a determined fight against the code by a class of men, who, at least in their own opinion, rank high above the common herd, as they are pleased to call us. According to these distinguished gentlemen, our code is useless, old fashioned rubbish, which can not be swept away any too soon, as gentlemen require no code to govern their conduct. Carry this line of argument a little further and to be consistent you will have to abolish every code, whether it be that of morality, religion or law, and allow each individual to decide for himself what is right and what is wrong.

However, do not for one moment delude yourselves into thinking that these medical anarchists do not believe in any code. They are the most fanatical worshippers of their chosen code —the code of the almighty dollar. They would sell their birthrights for a mess of pottage and prostitute their professional honor for a paltry fee. Upon the prospect of a consultation fee you will find them ever willing to meet homeopaths, eclectics, abortionists and quacks of all descriptions in consultation. In consultations with physicians you will find them always ready to agree with you in the consulting room, but to the family express in a gentle and tender manner their misgivings of your management of the case, and perchance in a little while you may find them in charge of the patient, particularly if you are a young physician without influence or friends. No opportunity is missed by them to see their names in the daily prints, always anxious to be interviewed on any subject, the source of considerable revenue to many an impecunious newspaper reporter. You may look for their names in testimonials endorsing anything under the sun, from salad dressings to yosilante underwear, anything, everything, provided only the testimonial be given wide circulation, especially in the daily press, with all the titles in full.

These gentlemen have a profound contempt for the code of ethics—they want none of it, and with good reason—no criminal ever admired the law that hung him.

Gentlemen, the calling of a physician is an arduous one at best, trials and tribulations abound on every side—joys and pleasures are few and far between—but it is in the power of each and every one of us by following our guidon, the code of ethics, and maintaining its principles to the end, to have the happy consciousness of a life well spent. I know of no greater eulogy to the departed physician, than is comprised in these few words: "He lived up to the code."

